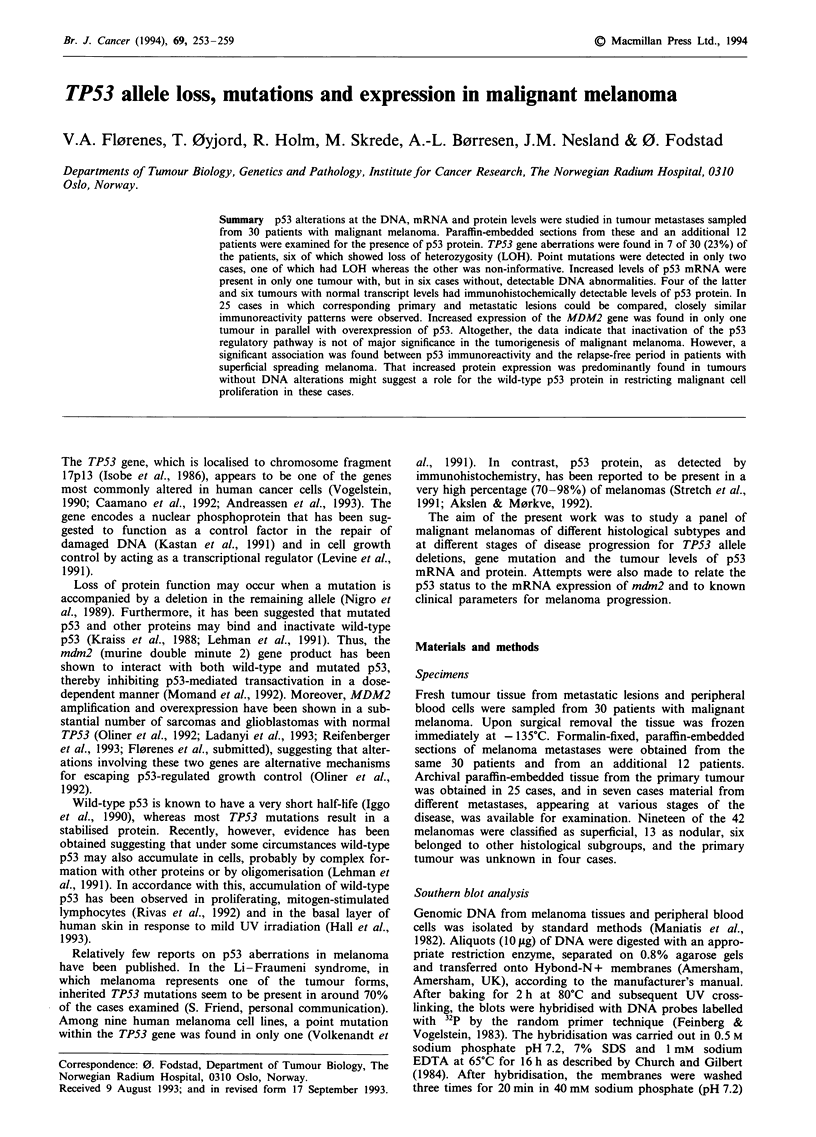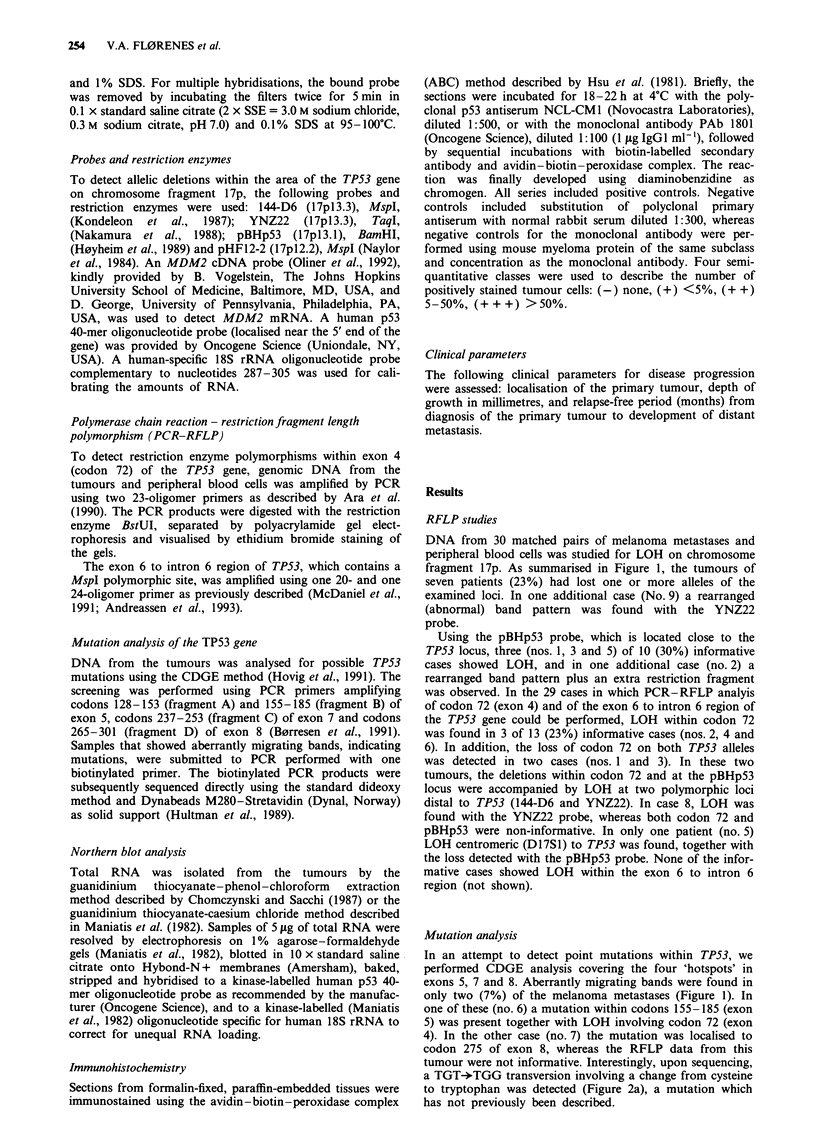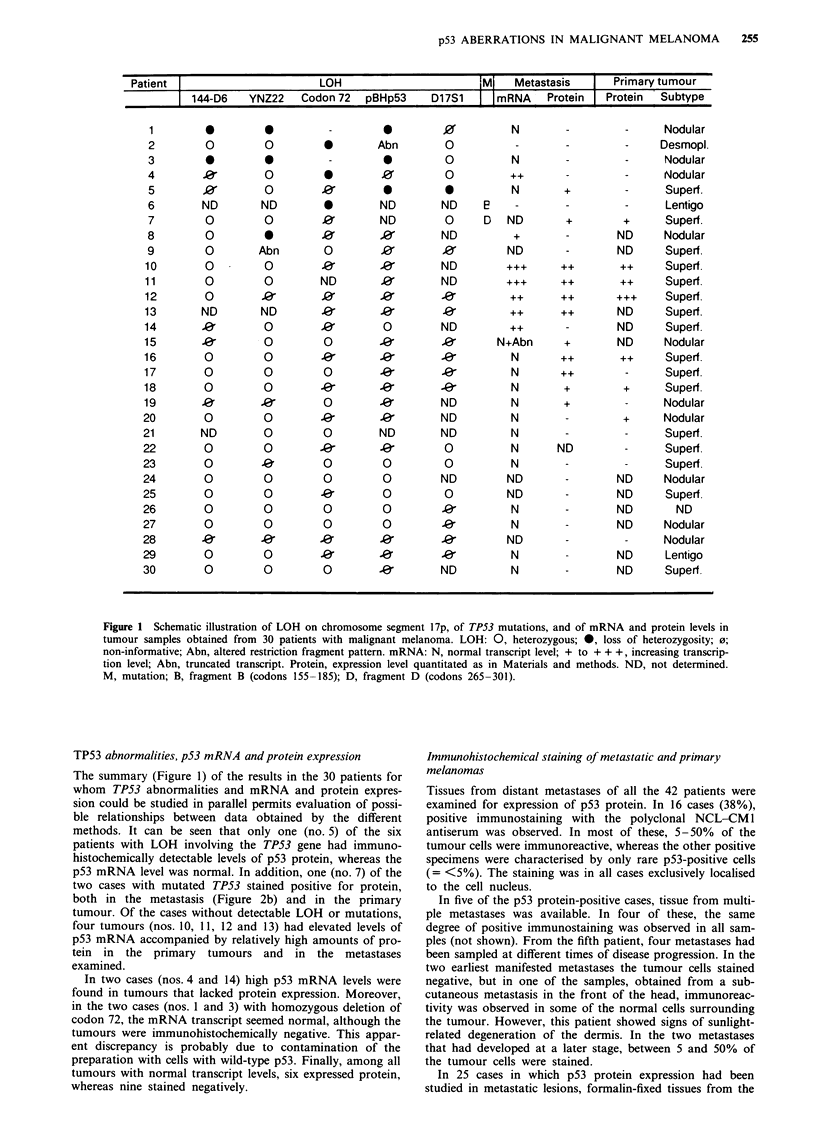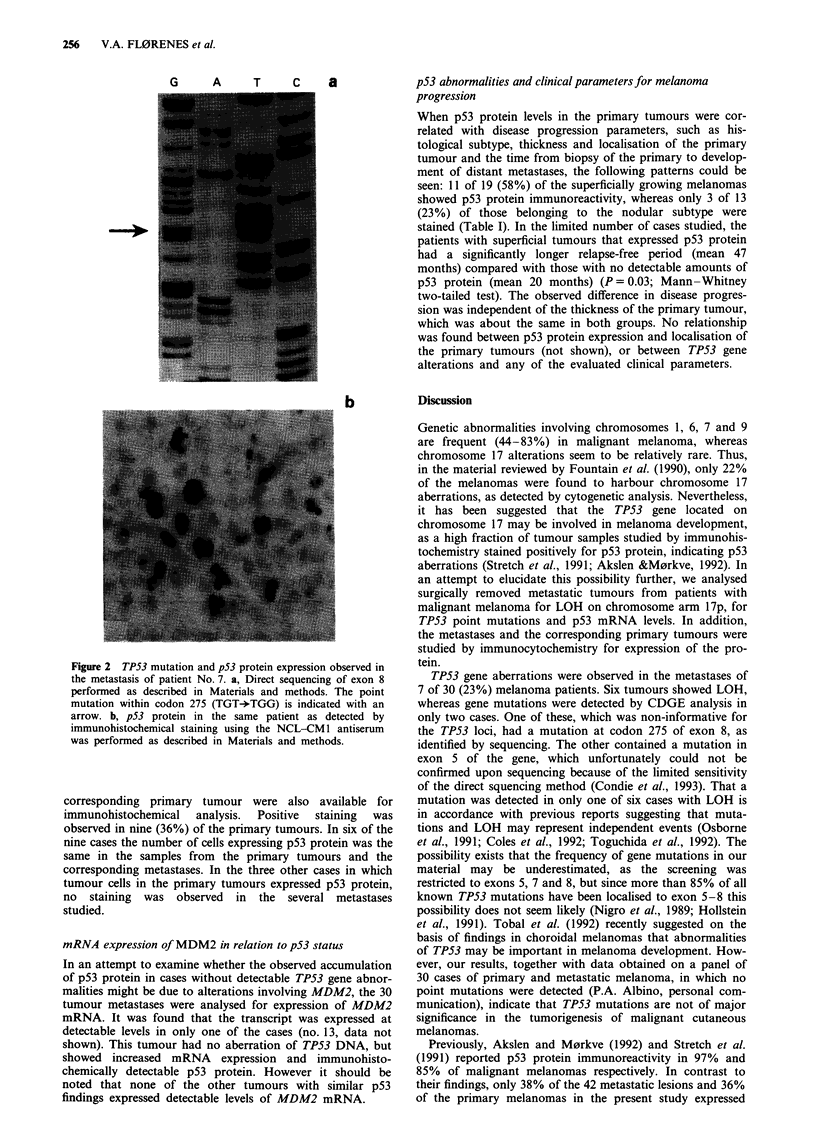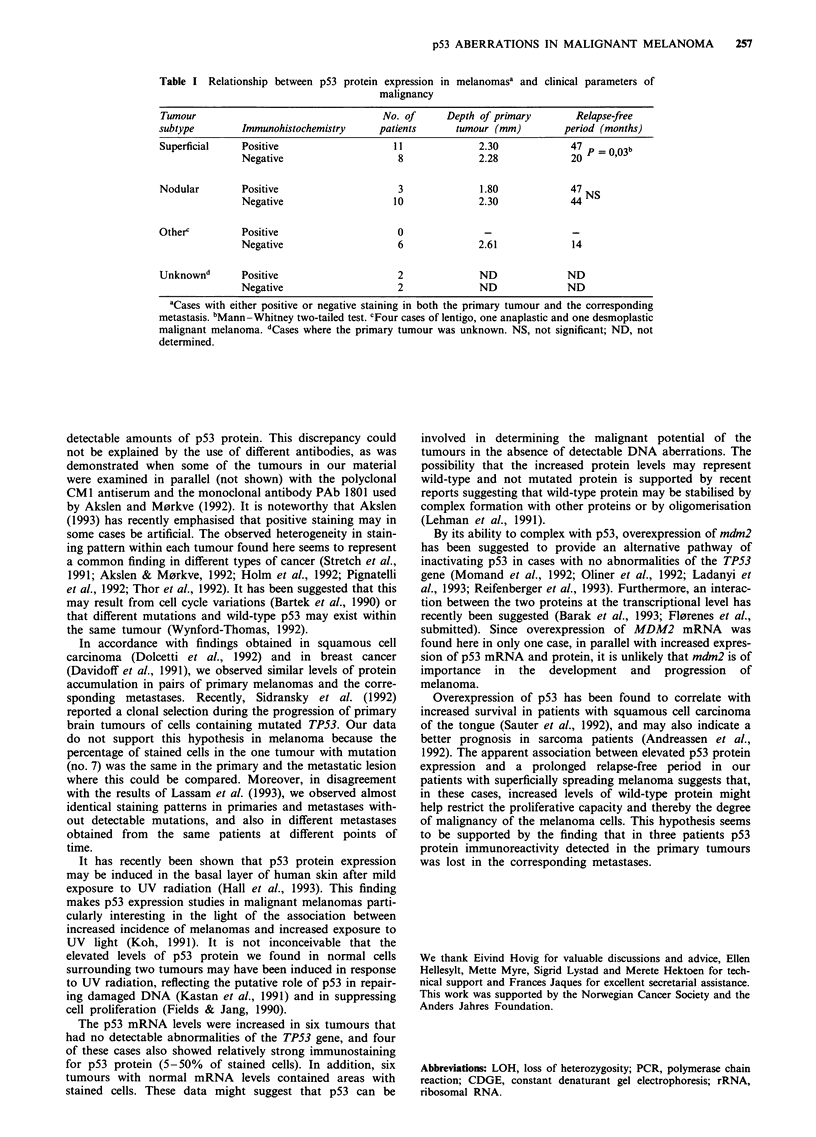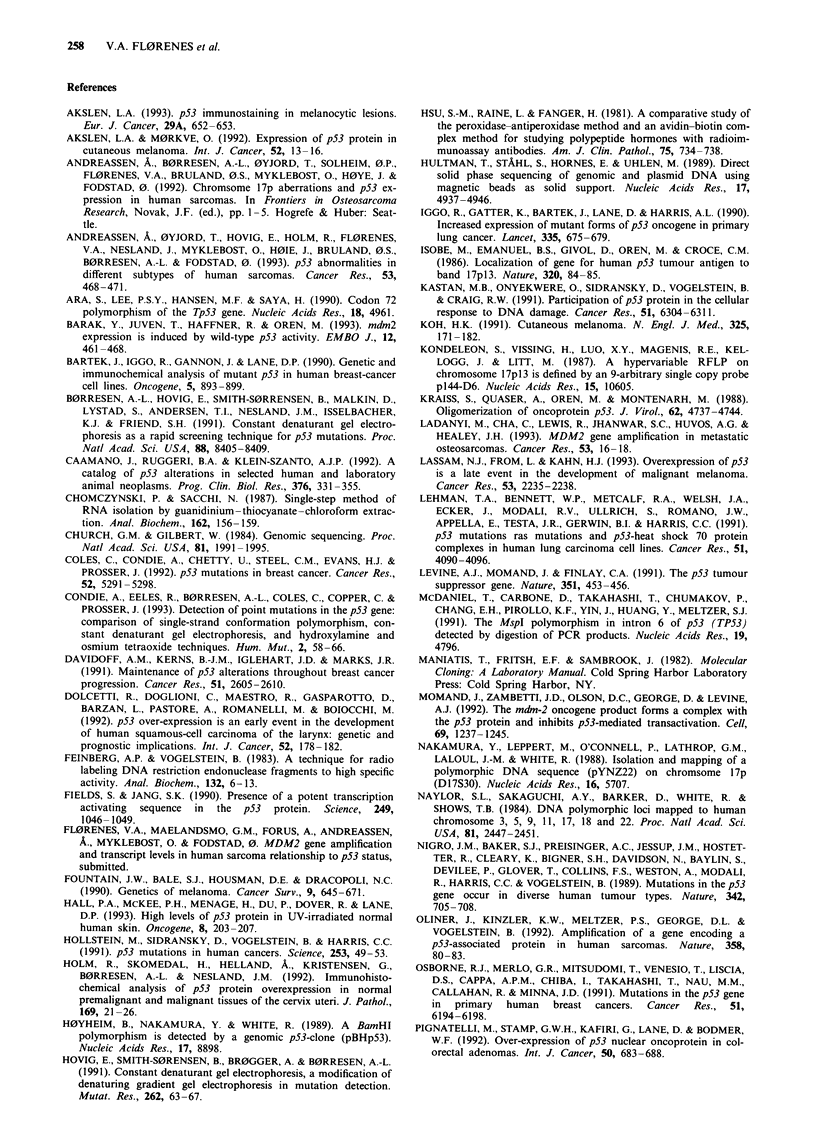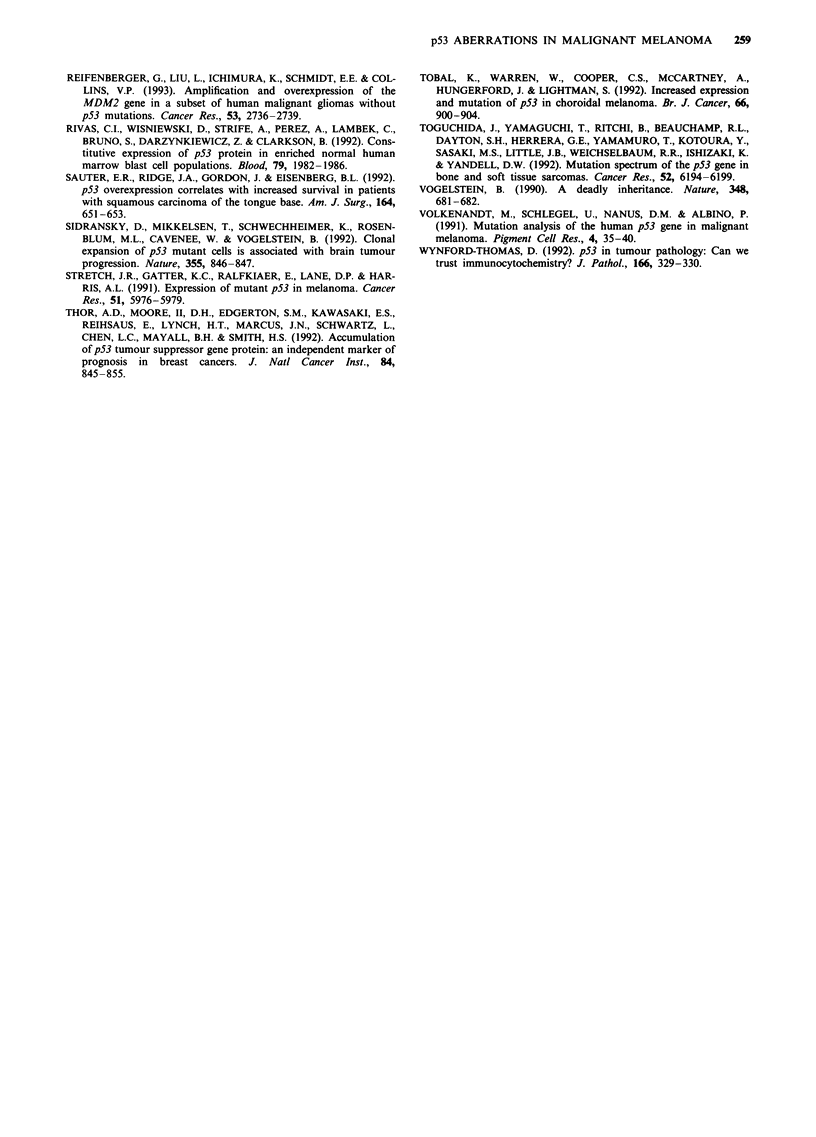# TP53 allele loss, mutations and expression in malignant melanoma.

**DOI:** 10.1038/bjc.1994.48

**Published:** 1994-02

**Authors:** V. A. Flørenes, T. Oyjord, R. Holm, M. Skrede, A. L. Børresen, J. M. Nesland, O. Fodstad

**Affiliations:** Department of Tumour Biology, Norwegian Radium Hospital, Oslo.

## Abstract

**Images:**


Br. J. Cancer (1994), 69, 253–259 © Macmillan Press Ltd., 1994

### *TP53* allele loss, mutations and expression in malignant melanoma

V.A. Flørenes, T. Øyjord, R. Holm, M. Skrede, A.-L. Børresen, J.M. Nesland & Ø. Fodstad

*Departments of Tumour Biology, Genetics and Pathology, Institute for Cancer Research, The Norwegian Radium Hospital, 0310 Oslo, Norway.*

**Summary** p53 alterations at the DNA, mRNA and protein levels were studied in tumour metastases sampled from 30 patients with malignant melanoma. Paraffin-embedded sections from these and an additional 12 patients were examined for the presence of p53 protein. *TP53* gene aberrations were found in 7 of 30 (23%) of the patients, six of which showed loss of heterozygosity (LOH). Point mutations were detected in only two cases, one of which had LOH whereas the other was non-informative. Increased levels of p53 mRNA were present in only one tumour with, but in six cases without, detectable DNA abnormalities. Four of the latter and six tumours with normal transcript levels had immunohistochemically detectable levels of p53 protein. In 25 cases in which corresponding primary and metastatic lesions could be compared, closely similar immunoreactivity patterns were observed. Increased expression of the *MDM2* gene was found in only one tumour in parallel with overexpression of p53. Altogether, the data indicate that inactivation of the p53 regulatory pathway is not of major significance in the tumorigenesis of malignant melanoma. However, a significant association was found between p53 immunoreactivity and the relapse-free period in patients with superficial spreading melanoma. That increased protein expression was predominantly found in tumours without DNA alterations might suggest a role for the wild-type p53 protein in restricting malignant cell proliferation in these cases.

The *TP53* gene, which is localised to chromosome fragment 17p13 (Isobe *et al.*, 1986), appears to be one of the genes most commonly altered in human cancer cells (Vogelstein, 1990; Caamano *et al.*, 1992; Andreassen *et al.*, 1993). The gene encodes a nuclear phosphoprotein that has been suggested to function as a control factor in the repair of damaged DNA (Kastan *et al.*, 1991) and in cell growth control by acting as a transcriptional regulator (Levine *et al.*, 1991).

Loss of protein function may occur when a mutation is accompanied by a deletion in the remaining allele (Nigro *et al.*, 1989). Furthermore, it has been suggested that mutated p53 and other proteins may bind and inactivate wild-type p53 (Kraiss *et al.*, 1988; Lehman *et al.*, 1991). Thus, the *mdm2* (murine double minute 2) gene product has been shown to interact with both wild-type and mutated p53, thereby inhibiting p53-mediated transactivation in a dose-dependent manner (Momand *et al.*, 1992). Moreover, *MDM2* amplification and overexpression have been shown in a substantial number of sarcomas and glioblastomas with normal *TP53* (Oliner *et al.*, 1992; Ladanyi *et al.*, 1993; Reifenberger *et al.*, 1993; Flørenes *et al.*, submitted), suggesting that alterations involving these two genes are alternative mechanisms for escaping p53-regulated growth control (Oliner *et al.*, 1992).

Wild-type p53 is known to have a very short half-life (Iggo *et al.*, 1990), whereas most *TP53* mutations result in a stabilised protein. Recently, however, evidence has been obtained suggesting that under some circumstances wild-type p53 may also accumulate in cells, probably by complex formation with other proteins or by oligomerisation (Lehman *et al.*, 1991). In accordance with this, accumulation of wild-type p53 has been observed in proliferating, mitogen-stimulated lymphocytes (Rivas *et al.*, 1992) and in the basal layer of human skin in response to mild UV irradiation (Hall *et al.*, 1993).

Relatively few reports on p53 aberrations in melanoma have been published. In the Li–Fraumeni syndrome, in which melanoma represents one of the tumour forms, inherited *TP53* mutations seem to be present in around 70% of the cases examined (S. Friend, personal communication). Among nine human melanoma cell lines, a point mutation within the *TP53* gene was found in only one (Volkenandt *et al.*, 1991). In contrast, p53 protein, as detected by immunohistochemistry, has been reported to be present in a very high percentage (70–98%) of melanomas (Stretch *et al.*, 1991; Akslen & Mørkve, 1992).

The aim of the present work was to study a panel of malignant melanomas of different histological subtypes and at different stages of disease progression for *TP53* allele deletions, gene mutation and the tumour levels of p53 mRNA and protein. Attempts were also made to relate the p53 status to the mRNA expression of *mdm2* and to known clinical parameters for melanoma progression.

### Materials and methods

### *Specimens*

Fresh tumour tissue from metastatic lesions and peripheral blood cells were sampled from 30 patients with malignant melanoma. Upon surgical removal the tissue was frozen immediately at $-135$°C. Formalin-fixed, paraffin-embedded sections of melanoma metastases were obtained from the same 30 patients and from an additional 12 patients. Archival paraffin-embedded tissue from the primary tumour was obtained in 25 cases, and in seven cases material from different metastases, appearing at various stages of the disease, was available for examination. Nineteen of the 42 melanomas were classified as superficial, 13 as nodular, six belonged to other histological subgroups, and the primary tumour was unknown in four cases.

### *Southern blot analysis*

Genomic DNA from melanoma tissues and peripheral blood cells was isolated by standard methods (Maniatis *et al.*, 1982). Aliquots (10 µg) of DNA were digested with an appropriate restriction enzyme, separated on 0.8% agarose gels and transferred onto Hybond-N+ membranes (Amersham, Amersham, UK), according to the manufacturer's manual. After baking for 2 h at 80°C and subsequent UV cross-linking, the blots were hybridised with DNA probes labelled with $^{32}P$ by the random primer technique (Feinberg & Vogelstein, 1983). The hybridisation was carried out in 0.5 M sodium phosphate pH 7.2, 7% SDS and 1 mM sodium EDTA at 65°C for 16 h as described by Church and Gilbert (1984). After hybridisation, the membranes were washed three times for 20 min in 40 mM sodium phosphate (pH 7.2)

Correspondence: Ø. Fodstad, Department of Tumour Biology, The Norwegian Radium Hospital, 0310 Oslo, Norway.
Received 9 August 1993; and in revised form 17 September 1993.

and 1% SDS. For multiple hybridisations, the bound probe was removed by incubating the filters twice for 5 min in 0.1 × standard saline citrate (2 × SSE = 3.0 M sodium chloride, 0.3 M sodium citrate, pH 7.0) and 0.1% SDS at 95–100°C.

*Probes and restriction enzymes*

To detect allelic deletions within the area of the *TP53* gene on chromosome fragment 17p, the following probes and restriction enzymes were used: 144-D6 (17p13.3), *Msp*I, (Kondeleon *et al.*, 1987); YNZ22 (17p13.3), *Taq*I, (Nakamura *et al.*, 1988); pBHp53 (17p13.1), *Bam*HI, (Høyheim *et al.*, 1989) and pHF12-2 (17p12.2), *Msp*I (Naylor *et al.*, 1984). An *MDM2* cDNA probe (Oliner et *al.*, 1992), kindly provided by B. Vogelstein, The Johns Hopkins University School of Medicine, Baltimore, MD, USA, and D. George, University of Pennsylvania, Philadelphia, PA, USA, was used to detect *MDM2* mRNA. A human p53 40-mer oligonucleotide probe (localised near the 5′ end of the gene) was provided by Oncogene Science (Uniondale, NY, USA). A human-specific 18S rRNA oligonucleotide probe complementary to nucleotides 287–305 was used for calibrating the amounts of RNA.

*Polymerase chain reaction – restriction fragment length polymorphism (PCR-RFLP)*

To detect restriction enzyme polymorphisms within exon 4 (codon 72) of the *TP53* gene, genomic DNA from the tumours and peripheral blood cells was amplified by PCR using two 23-oligomer primers as described by Ara *et al.* (1990). The PCR products were digested with the restriction enzyme *Bst*UI, separated by polyacrylamide gel electrophoresis and visualised by ethidium bromide staining of the gels.

The exon 6 to intron 6 region of *TP53*, which contains a *Msp*I polymorphic site, was amplified using one 20- and one 24-oligomer primer as previously described (McDaniel *et al.*, 1991; Andreassen *et al.*, 1993).

*Mutation analysis of the* TP53 *gene*

DNA from the tumours was analysed for possible *TP53* mutations using the CDGE method (Hovig *et al.*, 1991). The screening was performed using PCR primers amplifying codons 128–153 (fragment A) and 155–185 (fragment B) of exon 5, codons 237–253 (fragment C) of exon 7 and codons 265–301 (fragment D) of exon 8 (Børresen *et al.*, 1991). Samples that showed aberrantly migrating bands, indicating mutations, were submitted to PCR performed with one biotinylated primer. The biotinylated PCR products were subsequently sequenced directly using the standard dideoxy method and Dynabeads M280–Stretavidin (Dynal, Norway) as solid support (Hultman *et al.*, 1989).

*Northern blot analysis*

Total RNA was isolated from the tumours by the guanidinium thiocyanate–phenol–chloroform extraction method described by Chomczynski and Sacchi (1987) or the guanidinium thiocyanate-caesium chloride method described in Maniatis *et al.* (1982). Samples of 5 μg of total RNA were resolved by electrophoresis on 1% agarose–formaldehyde gels (Maniatis *et al.*, 1982), blotted in 10 × standard saline citrate onto Hybond-N+ membranes (Amersham), baked, stripped and hybridised to a kinase-labelled human p53 40-mer oligonucleotide probe as recommended by the manufacturer (Oncogene Science), and to a kinase-labelled (Maniatis *et al.*, 1982) oligonucleotide specific for human 18S rRNA to correct for unequal RNA loading.

*Immunohistochemistry*

Sections from formalin-fixed, paraffin-embedded tissues were immunostained using the avidin–biotin–peroxidase complex (ABC) method described by Hsu *et al.* (1981). Briefly, the sections were incubated for 18–22 h at 4°C with the polyclonal p53 antiserum NCL-CM1 (Novocastra Laboratories), diluted 1:500, or with the monoclonal antibody PAb 1801 (Oncogene Science), diluted 1:100 (1 μg IgG1 ml$^{-1}$), followed by sequential incubations with biotin-labelled secondary antibody and avidin–biotin–peroxidase complex. The reaction was finally developed using diaminobenzidine as chromogen. All series included positive controls. Negative controls included substitution of polyclonal primary antiserum with normal rabbit serum diluted 1:300, whereas negative controls for the monoclonal antibody were performed using mouse myeloma protein of the same subclass and concentration as the monoclonal antibody. Four semi-quantitative classes were used to describe the number of positively stained tumour cells: (−) none, (+) <5%, (+ +) 5–50%, (+ + +) >50%.

*Clinical parameters*

The following clinical parameters for disease progression were assessed: localisation of the primary tumour, depth of growth in millimetres, and relapse-free period (months) from diagnosis of the primary tumour to development of distant metastasis.

### Results

*RFLP studies*

DNA from 30 matched pairs of melanoma metastases and peripheral blood cells was studied for LOH on chromosome fragment 17p. As summarised in Figure 1, the tumours of seven patients (23%) had lost one or more alleles of the examined loci. In one additional case (No. 9) a rearranged (abnormal) band pattern was found with the YNZ22 probe.

Using the pBHp53 probe, which is located close to the *TP53* locus, three (nos. 1, 3 and 5) of 10 (30%) informative cases showed LOH, and in one additional case (no. 2) a rearranged band pattern plus an extra restriction fragment was observed. In the 29 cases in which PCR–RFLP analyis of codon 72 (exon 4) and of the exon 6 to intron 6 region of the *TP53* gene could be performed, LOH within codon 72 was found in 3 of 13 (23%) informative cases (nos. 2, 4 and 6). In addition, the loss of codon 72 on both *TP53* alleles was detected in two cases (nos. 1 and 3). In these two tumours, the deletions within codon 72 and at the pBHp53 locus were accompanied by LOH at two polymorphic loci distal to *TP53* (144-D6 and YNZ22). In case 8, LOH was found with the YNZ22 probe, whereas both codon 72 and pBHp53 were non-informative. In only one patient (no. 5) LOH centromeric (D17S1) to *TP53* was found, together with the loss detected with the pBHp53 probe. None of the informative cases showed LOH within the exon 6 to intron 6 region (not shown).

*Mutation analysis*

In an attempt to detect point mutations within *TP53*, we performed CDGE analysis covering the four 'hotspots' in exons 5, 7 and 8. Aberrantly migrating bands were found in only two (7%) of the melanoma metastases (Figure 1). In one of these (no. 6) a mutation within codons 155–185 (exon 5) was present together with LOH involving codon 72 (exon 4). In the other case (no. 7) the mutation was localised to codon 275 of exon 8, whereas the RFLP data from this tumour were not informative. Interestingly, upon sequencing, a TGT→TGG transversion involving a change from cysteine to tryptophan was detected (Figure 2a), a mutation which has not previously been described.

| Patient | LOH | | | | | M | Metastasis | | Primary tumour | |
|---|---|---|---|---|---|---|---|---|---|---|
| | 144-D6 | YNZ22 | Codon 72 | pBHp53 | D17S1 | | mRNA | Protein | Protein | Subtype |
| 1 | ● | ● | - | ● | ø | | N | - | - | Nodular |
| 2 | O | O | ● | Abn | O | | - | - | - | Desmopl. |
| 3 | ● | ● | - | ● | O | | N | - | - | Nodular |
| 4 | ø | O | ● | ø | O | | ++ | - | - | Nodular |
| 5 | ø | O | ø | ● | ● | | N | + | - | Superf. |
| 6 | ND | ND | ● | ND | ND | B | - | - | - | Lentigo |
| 7 | O | O | ø | ND | O | D | ND | + | + | Superf. |
| 8 | O | ● | ø | ø | ND | | + | - | ND | Nodular |
| 9 | O | Abn | O | ø | ø | | ND | - | ND | Superf. |
| 10 | O | O | ø | ø | ND | | +++ | ++ | ++ | Superf. |
| 11 | O | O | ND | ø | ND | | +++ | ++ | ++ | Superf. |
| 12 | O | ø | ø | ø | ø | | ++ | ++ | +++ | Superf. |
| 13 | ND | ND | ø | ø | ø | | ++ | ++ | ND | Superf. |
| 14 | ø | O | ø | O | ND | | ++ | - | ND | Superf. |
| 15 | ø | O | O | ø | ø | | N+Abn | + | ND | Nodular |
| 16 | O | O | ø | ø | ø | | N | ++ | ++ | Superf. |
| 17 | O | O | O | ø | ø | | N | ++ | - | Superf. |
| 18 | O | O | ø | ø | ø | | N | + | + | Superf. |
| 19 | ø | ø | O | ø | ND | | N | + | - | Nodular |
| 20 | O | O | ø | ø | ND | | N | - | + | Nodular |
| 21 | ND | O | O | ND | ND | | N | - | - | Superf. |
| 22 | O | O | ø | ø | O | | N | ND | - | Superf. |
| 23 | O | ø | O | O | O | | N | - | - | Superf. |
| 24 | O | O | O | O | ND | | ND | - | ND | Nodular |
| 25 | O | O | ø | O | O | | ND | - | ND | Superf. |
| 26 | O | O | O | O | ø | | N | - | ND | ND |
| 27 | O | O | O | O | ø | | N | - | ND | Nodular |
| 28 | ø | ø | ø | ø | ø | | ND | - | - | Nodular |
| 29 | O | O | ø | ø | ø | | N | - | ND | Lentigo |
| 30 | O | O | O | ø | ND | | N | - | ND | Superf. |

**Figure 1** Schematic illustration of LOH on chromosome segment 17p, of *TP53* mutations, and of mRNA and protein levels in tumour samples obtained from 30 patients with malignant melanoma. LOH: O, heterozygous; ●, loss of heterozygosity; ø; non-informative; Abn, altered restriction fragment pattern. mRNA: N, normal transcript level; + to + + +, increasing transcription level; Abn, truncated transcript. Protein, expression level quantitated as in Materials and methods. ND, not determined. M, mutation; B, fragment B (codons 155–185); D, fragment D (codons 265–301).

### TP53 *abnormalities, p53 mRNA and protein expression*

The summary (Figure 1) of the results in the 30 patients for whom *TP53* abnormalities and mRNA and protein expression could be studied in parallel permits evaluation of possible relationships between data obtained by the different methods. It can be seen that only one (no. 5) of the six patients with LOH involving the *TP53* gene had immunohistochemically detectable levels of p53 protein, whereas the p53 mRNA level was normal. In addition, one (no. 7) of the two cases with mutated *TP53* stained positive for protein, both in the metastasis (Figure 2b) and in the primary tumour. Of the cases without detectable LOH or mutations, four tumours (nos. 10, 11, 12 and 13) had elevated levels of p53 mRNA accompanied by relatively high amounts of protein in the primary tumours and in the metastases examined.

In two cases (nos. 4 and 14) high p53 mRNA levels were found in tumours that lacked protein expression. Moreover, in the two cases (nos. 1 and 3) with homozygous deletion of codon 72, the mRNA transcript seemed normal, although the tumours were immunohistochemically negative. This apparent discrepancy is probably due to contamination of the preparation with cells with wild-type p53. Finally, among all tumours with normal transcript levels, six expressed protein, whereas nine stained negatively.

### *Immunohistochemical staining of metastatic and primary melanomas*

Tissues from distant metastases of all the 42 patients were examined for expression of p53 protein. In 16 cases (38%), positive immunostaining with the polyclonal NCL–CM1 antiserum was observed. In most of these, 5–50% of the tumour cells were immunoreactive, whereas the other positive specimens were characterised by only rare p53-positive cells (= <5%). The staining was in all cases exclusively localised to the cell nucleus.

In five of the p53 protein-positive cases, tissue from multiple metastases was available. In four of these, the same degree of positive immunostaining was observed in all samples (not shown). From the fifth patient, four metastases had been sampled at different times of disease progression. In the two earliest manifested metastases the tumour cells stained negative, but in one of the samples, obtained from a subcutaneous metastasis in the front of the head, immunoreactivity was observed in some of the normal cells surrounding the tumour. However, this patient showed signs of sunlight-related degeneration of the dermis. In the two metastases that had developed at a later stage, between 5 and 50% of the tumour cells were stained.

In 25 cases in which p53 protein expression had been studied in metastatic lesions, formalin-fixed tissues from the

**Figure 2** *TP53* mutation and *p53* protein expression observed in the metastasis of patient No. 7. **a**, Direct sequencing of exon 8 performed as described in Materials and methods. The point mutation within codon 275 (TGT→TGG) is indicated with an arrow. **b**, *p53* protein in the same patient as detected by immunohistochemical staining using the NCL-CM1 antiserum was performed as described in Materials and methods.

corresponding primary tumour were also available for immunohistochemical analysis. Positive staining was observed in nine (36%) of the primary tumours. In six of the nine cases the number of cells expressing p53 protein was the same in the samples from the primary tumours and the corresponding metastases. In the three other cases in which tumour cells in the primary tumours expressed p53 protein, no staining was observed in the several metastases studied.

*mRNA expression of* MDM2 *in relation to p53 status*

In an attempt to examine whether the observed accumulation of p53 protein in cases without detectable *TP53* gene abnormalities might be due to alterations involving *MDM2*, the 30 tumour metastases were analysed for expression of *MDM2* mRNA. It was found that the transcript was expressed at detectable levels in only one of the cases (no. 13, data not shown). This tumour had no aberration of *TP53* DNA, but showed increased mRNA expression and immunohistochemically detectable p53 protein. However it should be noted that none of the other tumours with similar p53 findings expressed detectable levels of *MDM2* mRNA.

*p53 abnormalities and clinical parameters for melanoma progression*

When p53 protein levels in the primary tumours were correlated with disease progression parameters, such as histological subtype, thickness and localisation of the primary tumour and the time from biopsy of the primary to development of distant metastases, the following patterns could be seen: 11 of 19 (58%) of the superficially growing melanomas showed p53 protein immunoreactivity, whereas only 3 of 13 (23%) of those belonging to the nodular subtype were stained (Table I). In the limited number of cases studied, the patients with superficial tumours that expressed p53 protein had a significantly longer relapse-free period (mean 47 months) compared with those with no detectable amounts of p53 protein (mean 20 months) ($P = 0.03$; Mann-Whitney two-tailed test). The observed difference in disease progression was independent of the thickness of the primary tumour, which was about the same in both groups. No relationship was found between p53 protein expression and localisation of the primary tumours (not shown), or between *TP53* gene alterations and any of the evaluated clinical parameters.

### Discussion

Genetic abnormalities involving chromosomes 1, 6, 7 and 9 are frequent (44-83%) in malignant melanoma, whereas chromosome 17 alterations seem to be relatively rare. Thus, in the material reviewed by Fountain *et al.* (1990), only 22% of the melanomas were found to harbour chromosome 17 aberrations, as detected by cytogenetic analysis. Nevertheless, it has been suggested that the *TP53* gene located on chromosome 17 may be involved in melanoma development, as a high fraction of tumour samples studied by immunohistochemistry stained positively for p53 protein, indicating p53 aberrations (Stretch *et al.*, 1991; Akslen &Mørkve, 1992). In an attempt to elucidate this possibility further, we analysed surgically removed metastatic tumours from patients with malignant melanoma for LOH on chromosome arm 17p, for *TP53* point mutations and p53 mRNA levels. In addition, the metastases and the corresponding primary tumours were studied by immunocytochemistry for expression of the protein.

*TP53* gene aberrations were observed in the metastases of 7 of 30 (23%) melanoma patients. Six tumours showed LOH, whereas gene mutations were detected by CDGE analysis in only two cases. One of these, which was non-informative for the *TP53* loci, had a mutation at codon 275 of exon 8, as identified by sequencing. The other contained a mutation in exon 5 of the gene, which unfortunately could not be confirmed upon sequencing because of the limited sensitivity of the direct squencing method (Condie *et al.*, 1993). That a mutation was detected in only one of six cases with LOH is in accordance with previous reports suggesting that mutations and LOH may represent independent events (Osborne *et al.*, 1991; Coles *et al.*, 1992; Toguchida *et al.*, 1992). The possibility exists that the frequency of gene mutations in our material may be underestimated, as the screening was restricted to exons 5, 7 and 8, but since more than 85% of all known *TP53* mutations have been localised to exon 5-8 this possibility does not seem likely (Nigro *et al.*, 1989; Hollstein *et al.*, 1991). Tobal *et al.* (1992) recently suggested on the basis of findings in choroidal melanomas that abnormalities of *TP53* may be important in melanoma development. However, our results, together with data obtained on a panel of 30 cases of primary and metastatic melanoma, in which no point mutations were detected (P.A. Albino, personal communication), indicate that *TP53* mutations are not of major significance in the tumorigenesis of malignant cutaneous melanomas.

Previously, Akslen and Mørkve (1992) and Stretch *et al.* (1991) reported p53 protein immunoreactivity in 97% and 85% of malignant melanomas respectively. In contrast to their findings, only 38% of the 42 metastatic lesions and 36% of the primary melanomas in the present study expressed

**Table I** Relationship between p53 protein expression in melanomas[a] and clinical parameters of malignancy

| *Tumour subtype* | *Immunohistochemistry* | *No. of patients* | *Depth of primary tumour (mm)* | *Relapse-free period (months)* |
|---|---|---|---|---|
| Superficial | Positive | 11 | 2.30 | 47 $P = 0{,}03$[b] |
| | Negative | 8 | 2.28 | 20 |
| Nodular | Positive | 3 | 1.80 | 47 NS |
| | Negative | 10 | 2.30 | 44 |
| Other[c] | Positive | 0 | – | – |
| | Negative | 6 | 2.61 | 14 |
| Unknown[d] | Positive | 2 | ND | ND |
| | Negative | 2 | ND | ND |

[a]Cases with either positive or negative staining in both the primary tumour and the corresponding metastasis. [b]Mann–Whitney two-tailed test. [c]Four cases of lentigo, one anaplastic and one desmoplastic malignant melanoma. [d]Cases where the primary tumour was unknown. NS, not significant; ND, not determined.

detectable amounts of p53 protein. This discrepancy could not be explained by the use of different antibodies, as was demonstrated when some of the tumours in our material were examined in parallel (not shown) with the polyclonal CM1 antiserum and the monoclonal antibody PAb 1801 used by Akslen and Mørkve (1992). It is noteworthy that Akslen (1993) has recently emphasised that positive staining may in some cases be artificial. The observed heterogeneity in staining pattern within each tumour found here seems to represent a common finding in different types of cancer (Stretch *et al.*, 1991; Akslen & Mørkve, 1992; Holm *et al.*, 1992; Pignatelli *et al.*, 1992; Thor *et al.*, 1992). It has been suggested that this may result from cell cycle variations (Bartek *et al.*, 1990) or that different mutations and wild-type p53 may exist within the same tumour (Wynford-Thomas, 1992).

In accordance with findings obtained in squamous cell carcinoma (Dolcetti *et al.*, 1992) and in breast cancer (Davidoff *et al.*, 1991), we observed similar levels of protein accumulation in pairs of primary melanomas and the corresponding metastases. Recently, Sidransky *et al.* (1992) reported a clonal selection during the progression of primary brain tumours of cells containing mutated *TP53*. Our data do not support this hypothesis in melanoma because the percentage of stained cells in the one tumour with mutation (no. 7) was the same in the primary and the metastatic lesion where this could be compared. Moreover, in disagreement with the results of Lassam *et al.* (1993), we observed almost identical staining patterns in primaries and metastases without detectable mutations, and also in different metastases obtained from the same patients at different points of time.

It has recently been shown that p53 protein expression may be induced in the basal layer of human skin after mild exposure to UV radiation (Hall *et al.*, 1993). This finding makes p53 expression studies in malignant melanomas particularly interesting in the light of the association between increased incidence of melanomas and increased exposure to UV light (Koh, 1991). It is not inconceivable that the elevated levels of p53 protein we found in normal cells surrounding two tumours may have been induced in response to UV radiation, reflecting the putative role of p53 in repairing damaged DNA (Kastan *et al.*, 1991) and in suppressing cell proliferation (Fields & Jang, 1990).

The p53 mRNA levels were increased in six tumours that had no detectable abnormalities of the *TP53* gene, and four of these cases also showed relatively strong immunostaining for p53 protein (5–50% of stained cells). In addition, six tumours with normal mRNA levels contained areas with stained cells. These data might suggest that p53 can be involved in determining the malignant potential of the tumours in the absence of detectable DNA aberrations. The possibility that the increased protein levels may represent wild-type and not mutated protein is supported by recent reports suggesting that wild-type protein may be stabilised by complex formation with other proteins or by oligomerisation (Lehman *et al.*, 1991).

By its ability to complex with p53, overexpression of *mdm2* has been suggested to provide an alternative pathway of inactivating p53 in cases with no abnormalities of the *TP53* gene (Momand *et al.*, 1992; Oliner *et al.*, 1992; Ladanyi *et al.*, 1993; Reifenberger *et al.*, 1993). Furthermore, an interaction between the two proteins at the transcriptional level has recently been suggested (Barak *et al.*, 1993; Flørenes *et al.*, submitted). Since overexpression of *MDM2* mRNA was found here in only one case, in parallel with increased expression of p53 mRNA and protein, it is unlikely that *mdm2* is of importance in the development and progression of melanoma.

Overexpression of p53 has been found to correlate with increased survival in patients with squamous cell carcinoma of the tongue (Sauter *et al.*, 1992), and may also indicate a better prognosis in sarcoma patients (Andreassen *et al.*, 1992). The apparent association between elevated p53 protein expression and a prolonged relapse-free period in our patients with superficially spreading melanoma suggests that, in these cases, increased levels of wild-type protein might help restrict the proliferative capacity and thereby the degree of malignancy of the melanoma cells. This hypothesis seems to be supported by the finding that in three patients p53 protein immunoreactivity detected in the primary tumours was lost in the corresponding metastases.

We thank Eivind Hovig for valuable discussions and advice, Ellen Hellesylt, Mette Myre, Sigrid Lystad and Merete Hektoen for technical support and Frances Jaques for excellent secretarial assistance. This work was supported by the Norwegian Cancer Society and the Anders Jahres Foundation.

**Abbreviations:** LOH, loss of heterozygosity; PCR, polymerase chain reaction; CDGE, constant denaturant gel electrophoresis; rRNA, ribosomal RNA.

### References

AKSLEN, L.A. (1993). *p53* immunostaining in melanocytic lesions. *Eur. J. Cancer*, **29A**, 652–653.

AKSLEN, L.A. & MØRKVE, O. (1992). Expression of *p53* protein in cutaneous melanoma. *Int. J. Cancer*, **52**, 13–16.

ANDREASSEN, Å., BØRRESEN, A.-L., ØYJORD, T., SOLHEIM, Ø.P., FLØRENES, V.A., BRULAND, Ø.S., MYKLEBOST, O., HØYE, J. & FODSTAD, Ø. (1992). Chromsome 17p aberrations and *p53* expression in human sarcomas. In *Frontiers in Osteosarcoma Research*, Novak, J.F. (ed.), pp. 1–5. Hogrefe & Huber: Seattle.

ANDREASSEN, Å., ØYJORD, T., HOVIG, E., HOLM, R., FLØRENES, V.A., NESLAND, J., MYKLEBOST, O., HØIE, J., BRULAND, Ø.S., BØRRESEN, A.-L. & FODSTAD, Ø. (1993). *p53* abnormalities in different subtypes of human sarcomas. *Cancer Res.*, **53**, 468–471.

ARA, S., LEE, P.S.Y., HANSEN, M.F. & SAYA, H. (1990). Codon 72 polymorphism of the *Tp53* gene. *Nucleic Acids Res.*, **18**, 4961.

BARAK, Y., JUVEN, T., HAFFNER, R. & OREN, M. (1993). *mdm2* expression is induced by wild-type *p53* activity. *EMBO J.*, **12**, 461–468.

BARTEK, J., IGGO, R., GANNON, J. & LANE, D.P. (1990). Genetic and immunochemical analysis of mutant *p53* in human breast-cancer cell lines. *Oncogene*, **5**, 893–899.

BØRRESEN, A.-L., HOVIG, E., SMITH-SØRRENSEN, B., MALKIN, D., LYSTAD, S., ANDERSEN, T.I., NESLAND, J.M., ISSELBACHER, K.J. & FRIEND, S.H. (1991). Constant denaturant gel electrophoresis as a rapid screening technique for *p53* mutations. *Proc. Natl Acad. Sci. USA*, **88**, 8405–8409.

CAAMANO, J., RUGGERI, B.A. & KLEIN-SZANTO, A.J.P. (1992). A catalog of *p53* alterations in selected human and laboratory animal neoplasms. *Prog. Clin. Biol. Res.*, **376**, 331–355.

CHOMCZYNSKI, P. & SACCHI, N. (1987). Single-step method of RNA isolation by guanidinium–thiocyanate–chloroform extraction. *Anal. Biochem.*, **162**, 156–159.

CHURCH, G.M. & GILBERT, W. (1984). Genomic sequencing. *Proc. Natl Acad. Sci. USA*, **81**, 1991–1995.

COLES, C., CONDIE, A., CHETTY, U., STEEL, C.M., EVANS, H.J. & PROSSER, J. (1992). *p53* mutations in breast cancer. *Cancer Res.*, **52**, 5291–5298.

CONDIE, A., EELES, R., BØRRESEN, A.-L., COLES, C., COPPER, C. & PROSSER, J. (1993). Detection of point mutations in the *p53* gene: comparison of single-strand conformation polymorphism, constant denaturant gel electrophoresis, and hydroxylamine and osmium tetraoxide techniques. *Hum. Mut.*, **2**, 58–66.

DAVIDOFF, A.M., KERNS, B.-J.M., IGLEHART, J.D. & MARKS, J.R. (1991). Maintenance of *p53* alterations throughout breast cancer progression. *Cancer Res.*, **51**, 2605–2610.

DOLCETTI, R., DOGLIONI, C., MAESTRO, R., GASPAROTTO, D., BARZAN, L., PASTORE, A., ROMANELLI, M. & BOIOCCHI, M. (1992). *p53* over-expression is an early event in the development of human squamous-cell carcinoma of the larynx: genetic and prognostic implications. *Int. J. Cancer*, **52**, 178–182.

FEINBERG, A.P. & VOGELSTEIN, B. (1983). A technique for radio labeling DNA restriction endonuclease fragments to high specific activity. *Anal. Biochem.*, **132**, 6–13.

FIELDS, S. & JANG, S.K. (1990). Presence of a potent transcription activating sequence in the *p53* protein. *Science*, **249**, 1046–1049.

FLØRENES, V.A., MAELANDSMO, G.M., FORUS, A., ANDREASSEN, Å., MYKLEBOST, O. & FODSTAD, Ø. *MDM2* gene amplification and transcript levels in human sarcoma relationship to *p53* status, submitted.

FOUNTAIN, J.W., BALE, S.J., HOUSMAN, D.E. & DRACOPOLI, N.C. (1990). Genetics of melanoma. *Cancer Surv.*, **9**, 645–671.

HALL, P.A., McKEE, P.H., MENAGE, H., DU, P., DOVER, R. & LANE, D.P. (1993). High levels of *p53* protein in UV-irradiated normal human skin. *Oncogene*, **8**, 203–207.

HOLLSTEIN, M., SIDRANSKY, D., VOGELSTEIN, B. & HARRIS, C.C. (1991). *p53* mutations in human cancers. *Science*, **253**, 49–53.

HOLM, R., SKOMEDAL, H., HELLAND, Å., KRISTENSEN, G., BØRRESEN, A.-L. & NESLAND, J.M. (1992). Immunohistochemical analysis of *p53* protein overexpression in normal premalignant and malignant tissues of the cervix uteri. *J. Pathol.*, **169**, 21–26.

HØYHEIM, B., NAKAMURA, Y. & WHITE, R. (1989). A *Bam*HI polymorphism is detected by a genomic *p53*-clone (pBHp53). *Nucleic Acids Res.*, **17**, 8898.

HOVIG, E., SMITH-SØRENSEN, B., BRØGGER, A. & BØRRESEN, A.-L. (1991). Constant denaturant gel electrophoresis, a modification of denaturing gradient gel electrophoresis in mutation detection. *Mutat. Res.*, **262**, 63–67.

HSU, S.-M., RAINE, L. & FANGER, H. (1981). A comparative study of the peroxidase–antiperoxidase method and an avidin–biotin complex method for studying polypeptide hormones with radioimmunoassay antibodies. *Am. J. Clin. Pathol.*, **75**, 734–738.

HULTMAN, T., STÅHL, S., HORNES, E. & UHLEN, M. (1989). Direct solid phase sequencing of genomic and plasmid DNA using magnetic beads as solid support. *Nucleic Acids Res.*, **17**, 4937–4946.

IGGO, R., GATTER, K., BARTEK, J., LANE, D. & HARRIS, A.L. (1990). Increased expression of mutant forms of *p53* oncogene in primary lung cancer. *Lancet*, **335**, 675–679.

ISOBE, M., EMANUEL, B.S., GIVOL, D., OREN, M. & CROCE, C.M. (1986). Localization of gene for human *p53* tumour antigen to band 17p13. *Nature*, **320**, 84–85.

KASTAN, M.B., ONYEKWERE, O., SIDRANSKY, D., VOGELSTEIN, B. & CRAIG, R.W. (1991). Participation of *p53* protein in the cellular response to DNA damage. *Cancer Res.*, **51**, 6304–6311.

KOH, H.K. (1991). Cutaneous melanoma. *N. Engl. J. Med.*, **325**, 171–182.

KONDELEON, S., VISSING, H., LUO, X.Y., MAGENIS, R.E., KELLOGG, J. & LITT, M. (1987). A hypervariable RFLP on chromosome 17p13 is defined by an 9-arbitrary single copy probe p144-D6. *Nucleic Acids Res.*, **15**, 10605.

KRAISS, S., QUASER, A., OREN, M. & MONTENARH, M. (1988). Oligomerization of oncoprotein *p53*. *J. Virol.*, **62**, 4737–4744.

LADANYI, M., CHA, C., LEWIS, R., JHANWAR, S.C., HUVOS, A.G. & HEALEY, J.H. (1993). *MDM2* gene amplification in metastatic osteosarcomas. *Cancer Res.*, **53**, 16–18.

LASSAM, N.J., FROM, L. & KAHN, H.J. (1993). Overexpression of *p53* is a late event in the development of malignant melanoma. *Cancer Res.*, **53**, 2235–2238.

LEHMAN, T.A., BENNETT, W.P., METCALF, R.A., WELSH, J.A., ECKER, J., MODALI, R.V., ULLRICH, S., ROMANO, J.W., APPELLA, E., TESTA, J.R., GERWIN, B.I. & HARRIS, C.C. (1991). *p53* mutations ras mutations and *p53*-heat shock 70 protein complexes in human lung carcinoma cell lines. *Cancer Res.*, **51**, 4090–4096.

LEVINE, A.J., MOMAND, J. & FINLAY, C.A. (1991). The *p53* tumour suppressor gene. *Nature*, **351**, 453–456.

McDANIEL, T., CARBONE, D., TAKAHASHI, T., CHUMAKOV, P., CHANG, E.H., PIROLLO, K.F., YIN, J., HUANG, Y., MELTZER, S.J. (1991). The *Msp*I polymorphism in intron 6 of *p53 (TP53)* detected by digestion of PCR products. *Nucleic Acids Res.*, **19**, 4796.

MANIATIS, T., FRITSH, E.F. & SAMBROOK, J. (1982). *Molecular Cloning: A Laboratory Manual.* Cold Spring Harbor Laboratory Press: Cold Spring Harbor, NY.

MOMAND, J., ZAMBETTI, J.D., OLSON, D.C., GEORGE, D. & LEVINE, A.J. (1992). The *mdm-2* oncogene product forms a complex with the *p53* protein and inhibits *p53*-mediated transactivation. *Cell*, **69**, 1237–1245.

NAKAMURA, Y., LEPPERT, M., O'CONNELL, P., LATHROP, G.M., LALOUL, J.-M. & WHITE, R. (1988). Isolation and mapping of a polymorphic DNA sequence (pYNZ22) on chromsome 17p (D17S30). *Nucleic Acids Res.*, **16**, 5707.

NAYLOR, S.L., SAKAGUCHI, A.Y., BARKER, D., WHITE, R. & SHOWS, T.B. (1984). DNA polymorphic loci mapped to human chromosome 3, 5, 9, 11, 17, 18 and 22. *Proc. Natl Acad. Sci. USA*, **81**, 2447–2451.

NIGRO, J.M., BAKER, S.J., PREISINGER, A.C., JESSUP, J.M., HOSTETTER, R., CLEARY, K., BIGNER, S.H., DAVIDSON, N., BAYLIN, S., DEVILEE, P., GLOVER, T., COLLINS, F.S., WESTON, A., MODALI, R., HARRIS, C.C. & VOGELSTEIN, B. (1989). Mutations in the *p53* gene occur in diverse human tumour types. *Nature*, **342**, 705–708.

OLINER, J., KINZLER, K.W., MELTZER, P.S., GEORGE, D.L. & VOGELSTEIN, B. (1992). Amplification of a gene encoding a *p53*-associated protein in human sarcomas. *Nature*, **358**, 80–83.

OSBORNE, R.J., MERLO, G.R., MITSUDOMI, T., VENESIO, T., LISCIA, D.S., CAPPA, A.P.M., CHIBA, I., TAKAHASHI, T., NAU, M.M., CALLAHAN, R. & MINNA, J.D. (1991). Mutations in the *p53* gene in primary human breast cancers. *Cancer Res.*, **51**, 6194–6198.

PIGNATELLI, M., STAMP, G.W.H., KAFIRI, G., LANE, D. & BODMER, W.F. (1992). Over-expression of *p53* nuclear oncoprotein in colorectal adenomas. *Int. J. Cancer*, **50**, 683–688.

REIFENBERGER, G., LIU, L., ICHIMURA, K., SCHMIDT, E.E. & COLLINS, V.P. (1993). Amplification and overexpression of the *MDM2* gene in a subset of human malignant gliomas without *p53* mutations. *Cancer Res.*, **53**, 2736–2739.

RIVAS, C.I., WISNIEWSKI, D., STRIFE, A., PEREZ, A., LAMBEK, C., BRUNO, S., DARZYNKIEWICZ, Z. & CLARKSON, B. (1992). Constitutive expression of *p53* protein in enriched normal human marrow blast cell populations. *Blood*, **79**, 1982–1986.

SAUTER, E.R., RIDGE, J.A., GORDON, J. & EISENBERG, B.L. (1992). *p53* overexpression correlates with increased survival in patients with squamous carcinoma of the tongue base. *Am. J. Surg.*, **164**, 651–653.

SIDRANSKY, D., MIKKELSEN, T., SCHWECHHEIMER, K., ROSENBLUM, M.L., CAVENEE, W. & VOGELSTEIN, B. (1992). Clonal expansion of *p53* mutant cells is associated with brain tumour progression. *Nature*, **355**, 846–847.

STRETCH, J.R., GATTER, K.C., RALFKIAER, E., LANE, D.P. & HARRIS, A.L. (1991). Expression of mutant *p53* in melanoma. *Cancer Res.*, **51**, 5976–5979.

THOR, A.D., MOORE, II, D.H., EDGERTON, S.M., KAWASAKI, E.S., REIHSAUS, E., LYNCH, H.T., MARCUS, J.N., SCHWARTZ, L., CHEN, L.C., MAYALL, B.H. & SMITH, H.S. (1992). Accumulation of *p53* tumour suppressor gene protein: an independent marker of prognosis in breast cancers. *J. Natl Cancer Inst.*, **84**, 845–855.

TOBAL, K., WARREN, W., COOPER, C.S., MCCARTNEY, A., HUNGERFORD, J. & LIGHTMAN, S. (1992). Increased expression and mutation of *p53* in choroidal melanoma. *Br. J. Cancer*, **66**, 900–904.

TOGUCHIDA, J., YAMAGUCHI, T., RITCHI, B., BEAUCHAMP, R.L., DAYTON, S.H., HERRERA, G.E., YAMAMURO, T., KOTOURA, Y., SASAKI, M.S., LITTLE, J.B., WEICHSELBAUM, R.R., ISHIZAKI, K. & YANDELL, D.W. (1992). Mutation spectrum of the *p53* gene in bone and soft tissue sarcomas. *Cancer Res.*, **52**, 6194–6199.

VOGELSTEIN, B. (1990). A deadly inheritance. *Nature*, **348**, 681–682.

VOLKENANDT, M., SCHLEGEL, U., NANUS, D.M. & ALBINO, P. (1991). Mutation analysis of the human *p53* gene in malignant melanoma. *Pigment Cell Res.*, **4**, 35–40.

WYNFORD-THOMAS, D. (1992). *p53* in tumour pathology: Can we trust immunocytochemistry? *J. Pathol.*, **166**, 329–330.